# Current Status of Cardiac Rehabilitation in the Regional Cardiocerebrovascular Centers in Korea

**DOI:** 10.3390/jcm10215079

**Published:** 2021-10-29

**Authors:** Chul Kim, Jidong Sung, Jae-Young Han, Sungju Jee, Jang Woo Lee, Jong Hwa Lee, Won-Seok Kim, Heui Je Bang, Sora Baek, Kyung-Lim Joa, Ae Ryoung Kim, So Young Lee, Jihee Kim, Chung Reen Kim, Oh Pum Kwon

**Affiliations:** 1 Department of Rehabilitation Medicine, Inje University Sanggye Paik Hospital, Seoul 01757, Korea; 2 Division of Cardiology, Department of Medicine, Sungkyunkwan University School of Medicine, Seoul 06351, Korea; jidong.sung@gmail.com; 3 Department of Physical Medicine and Rehabilitation, Chonnam National University Medical School and Hospital, Gwangju 61469, Korea; rmhanjy@daum.net; 4 Department of Rehabilitation Medicine, Chungnam National University College of Medicine, Daejeon 35015, Korea; drjeesungju@cnuh.co; 5 Department of Physical Medicine and Rehabilitation, National Health Insurance Service Ilsan Hospital, Goyang 10444, Korea; medipia@gmail.com; 6 Department of Physical Medicine and Rehabilitation, Dong A University College of Medicine, Busan 49201, Korea; jhlee08@dau.ac.kr; 7 Department of Rehabilitation Medicine, Seoul National University Bundang Hospital, Seongnam 13620, Korea; Wondol77@gmail.com (W.-S.K.); Pum78@naver.com (O.P.K.); 8 Department of Rehabilitation Medicine, Chungbuk National University Hospital, Cheongju 28644, Korea; bang@chungbuk.ac.kr; 9 Department of Rehabilitation Medicine, Kangwon National University School of Medicine, Chuncheon 24289, Korea; sora.baek@kangwon.ac.kr; 10 Department of Rehabilitation Medicine, Inha University Hospital, Incheon 22332, Korea; drjoakl@gmail.com; 11 Department of Rehabilitation Medicine, School of Medicine Kyungpook National University, Daegu 41944, Korea; Ryoung20@hanmail.net; 12 Department of Rehabilitation Medicine, Jeju National University School of Medicine, Jeju 63241, Korea; bluelsy900@hanmail.net; 13 Department of Rehabilitation Medicine, Wonkwang University School of Medicine, Iksan 54538, Korea; gold82mouse@hanmail.net; 14 Department of Rehabilitation Medicine, Ulsan University College of Medicine, Ulsan 44033, Korea; crkim@uuh.ulsan.kr

**Keywords:** barriers, cardiac rehabilitation, hospital-based, participation rate

## Abstract

Regional Cardiocerebrovascular Centers (RCCs)—a Korean government initiative—seek to reduce medical gaps across regions, and their cardiac rehabilitation (CR) programs are expected to model post-acute care for the Korean CR program. Accordingly, this study aimed to evaluate the current status of CR programs in the RCCs. We distributed surveys on the CR condition, activity, and barriers to 12 RCCs in different provinces. The results revealed significant gaps in the annual number of acute myocardial infarction admissions, and CR candidates, capacity, and density across the 12 RCCs. The CR capacity (50–500) and density (0.42–7.36) indicated particularly large gaps. Twelve RCCs had the necessary facilities, equipment and personnel for CR assessments and management, with high CR referral (97%) and patient education (78%) rates. However, the inpatient CR exercise training (56%) participation rate was inadequate, with much lower enrollment (47%) and adherence (17%) rates to the outpatient CR program and large differences across centers. Therefore, this study’s results will provide the evidence required to establish special national health strategies to overcome the CR barriers of patient, doctor/hospital, and policy factors for activating Korean CR programs.

## 1. Introduction

Cardiovascular disease (CVD) is the leading cause of mortality, morbidity, and healthcare costs in South Korea [1]. Cardiovascular (CV) mortality has risen by 42.8% over the last decade. It has become the second leading cause of death in South Korea [2]. According to the 2019 statistical office data, there were 62.42 deaths due to CVD per 100,000 individuals [3]. The modifiable CV risk factors’ prevalence, such as smoking, dyslipidemia, obesity, and a sedentary lifestyle have continued to increase. The population is rapidly aging, with those aged 65 years and older projected to reach 37% by 2050 [1,4]. Death at the time of disease incidence, recurrence, and complications during follow-ups contribute to high mortality. There are also problems with decreased exercise capacity and quality of life. In addition, although most cases of CVD manifest clinically as an acute disease, they are actually chronic degenerative diseases that progress over a period of time. They thus need to be treated and managed as a chronic disease after discharge from the hospital [5].

According to the Organization for Economic Co-operation and Development (OECD) Reviews of Health Care Quality of Korea 2012, the “paradox” of South Korea was that case–fatality rates were higher for acute myocardial infarction (AMI) than for OECD countries [4]. This is despite having excellent acute hospital care. This was largely due to inadequate CVD risk factor prevention and suboptimal emergency response systems. Additional contributors are inadequate post-acute care and marked disparities between rural and urban communities. This “paradox” is likely to be reinforced by the insufficiency of cardiac rehabilitation (CR) services, leading to a higher number of readmitted patients [4].

**Figure 1:**
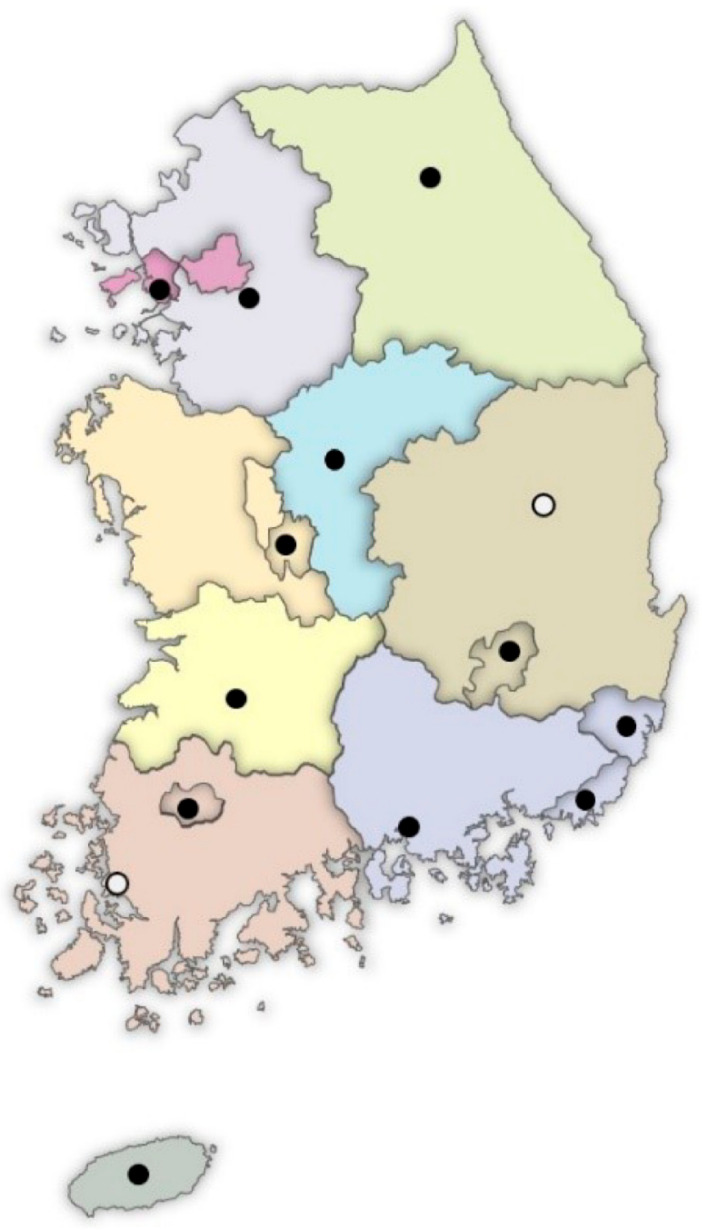
Locations of the Regional Cardiocerebrovascular Centers (RCCs) in South Korea. ● Twelve RCCs which are included in this study. ○ Two RCCs which were recently established and are not included in this study.

The Ministry of Health and Welfare (MHW) of South Korea has allocated government budgets from 2008 to install Regional Cardiocerebrovascular Centers (RCCs) in each province’s main cities, except for Seoul. Fourteen RCCs have been installed until the present (Figure 1). The RCC project’s goal was to minimize the incidence of complications and mortality. It also intends to reduce the medical gaps among the South Korean regions. This is conducted through timely medical service provision across the country. It also aims to facilitate patients’ earlier return to society after their complete recovery [6]. At the RCCs, standardized and high-quality care for AMI and stroke has been established. They are required to be equipped with CR facilities, equipment, and personnel. They also have regular performance measures conducted by the Korea Disease Control and Prevention Agency (KDCA) on behalf of the MHW (Table 1). As a result, the RCC project has led to improved outcomes and less acute fatality rates for patients who arrived within this implementation period. However, there were no significant changes in the overall in-hospital mortality [7]. Regarding post-acute care, the RCCs’ CR programs are expected to be a model for the Korean CR program. However, an evaluation study on their current status and achievements has not yet been reported. This study’s purpose is therefore to evaluate the current status of the CR programs in the RCCs. It also aims to make these data available for developing strategies to boost CR programs in Korea.

## 2. Materials and Methods

The study’s subjects were the RCCs in South Korea. We excluded the two newly designated RCCs (secondary hospitals) as the CR development processes were still ongoing. The CR program’s quality was also unsatisfactory as compared to that of existing RCCs, which are tertiary hospitals. A total of 12 RCCs participated in this study: Chonnam National University Hospital, Chungbuk National University Hospital, Chungnam National University Hospital, Dong-A University Hospital, Gyeongsang National University Hospital, Inha University Hospital, Jeju National University Hospital, Kangwon National University Hospital, Kyungpook National University Hospital, Seoul National University Bundang Hospital, Ulsan University Hospital, and Wonkwang University Hospital.

### 2.1. Development of CR Survey Forms

To examine CR’s current status in the 12 RCCs, the CR-General Questionnaire (CR-GQ) was developed after analyzing the national and international CR clinical practice guidelines (CPG) [5,8,9,10,11,12,13]. The Cardiac Rehabilitation-In Depth Questionnaire (CR-IDQ) was developed with reference to the CR evaluation tools of York University [14,15] and International Council of Cardiovascular Prevention and Rehabilitation (ICCPR; www.globalcardiacrehab.com, accessed on 9 September 2021) [16]. To analyze the current status of RCC, CR-GQ examined the: kinds of procedures offered, amount of procedures or surgeries, CR program’s initiation, and CR in the institution’s delivery system. CR-IDQ investigates the: CR components, CR capacity (patient capacity to be served each year in the institute), CR density, outpatient CR program’s commencement time, CR team personnel, facility, supervised exercise program, CPR certification, and further details on the outpatient CR program. Detailed information on CR-GQ and CR-IDQ are available in the Supplementary Materials thru online at https://www.mdpi.com/article/10.3390/jcm10215079/s1, Supplementary Materials S1: I. CR-GQ form, Supplementary Materials S2: II. CR-IDQ form.

### 2.2. Dispatch of the Surveys

Printed copies of the CR-GQ were sent to the staff of the cardiology, cardiac surgery, and rehabilitation medicine departments of the 12 RCCs. Printed copies of the CR-IDQ were sent to the staff responsible for CR in the 12 RCCs. The printed surveys, project information, and an explanation of how to respond to the survey were sent in July 2020. An official letter of cooperation from the Korea National Institute of Health was included to increase the response rate. The surveys’ responses were entered in a Google survey format that was accessible through a QR code provided with the package or by connecting to www.crsurvey.co.kr (accessed on 15 September 2020). The data were collected until 30 August 2020.

### 2.3. Confirmation of the Response

Due to the COVID-19 pandemic, the CR-GQ’s response rate was lower than expected. We initially planned to conduct a field survey if the response thereof was inadequate. However, field surveys were not possible as visitors’ access to hospitals was restricted due to the pandemic. Out of 44 responses to CR-GQ, the response rate was 59%. This excluded one case of duplicate response and cases with less than 50% completion. In all 12 RCCs, at least one response was received from the staff of the departments of cardiology and rehabilitation medicine. Responses from the staff of the cardiac surgeries were received from only six centers. All responses to the CR-IDQ were included in the study (*N* = 12, response rate 100%). The staff responsible for CR at each RCC reviewed and confirmed whether the number of candidates and CR participation rates in their RCCs from 1 July 2019, to 30 June 2020 were correct.

The overall performance achievement was evaluated based on the number of CR candidates, CR capacity, CR density, and CR participation rate. The number of CR candidates refers to the annual number of AMI admissions less the number of patients not indicated for CR referral. CR capacity refers to the median number of patients a program can serve annually. CR density refers to the annual number of CR candidates divided by the CR capacity [17]. The CR participation rate refers to the number of outpatient CR enrollments divided by the number of inpatient CR referrals.

### 2.4. Statistical Analysis

To determine whether the CR performance is different among RCCs, we compared the data collected via the surveys from CR directors at the 12 RCCs. An ANOVA was performed to compare CR candidates, CR capacity, and CR density. The chi-square test was used to compare the proportion of patients with CR participation rates. All statistical analyses were performed using the Statistical Package for the Social Sciences (SPSS) (version 19.0; SPSS Inc., Chicago, IL, USA). The level of significance was set at *p* < 0.05.

### 2.5. Ethics Statement

This study was approved by the Institutional Review Board of the Inje University Sanggye Paik Hospital, Korea (IRB No. SGPAIK202012010002-HE003). The need to obtain informed consent was waived due to the non-clinical nature of the study. No personal information (patient’s name, address, ID, phone number, hospital ID) were collected and thus the participants’ anonymity was preserved.

## 3. Results

The following results are based on the responses to CR-GQ and CR-IDQ from the 12 RCCs.

### 3.1. Number and Distribution of the CR Programs by Region in Korea

There were 164 hospitals (103 certified hospitals from the Korean Society of Interventional Cardiology), including the RCCs, which practiced percutaneous coronary intervention (PCI). Out of these 164, 47 (28.7%) had CR programs. There were regional gaps in the CR practice rate when considering the number of ischemic heart disease and AMI cases (Table 2). RCCs were the only hospitals to practice CR in the Daejeon-Chungnam, Chungbuk, Jeonbuk, Ulsan, and Jeju regions.

### 3.2. General Characteristics of 12 RCCs Related to the CR Program

CR programs were established in three RCCs from 2008 to 2009, three RCCs from 2010 to 2011, three RCCs from 2012 to 2013, two RCCs from 2016 to 2017, and three RCCs from 2019 to 2020. There was a total of 14 CR programs in practice. As previously mentioned, we excluded two newly designated RCCs (secondary hospitals) as their CR programs have not yet been established.

In the 12 RCCs, most CVD treatment procedures, such as: PCI, coronary artery bypass graft (CABG) surgery, acute management of heart failure, valvular surgery, percutaneous valve implantation, peripheral arterial stents and surgery, and implantation of pacemakers or implantable cardioverter defibrillators (ICDs), were practiced. However, left ventricular assist device (LVAD) implantation or heart transplants were performed in only seven centers. CR was performed for CABG surgery and angina without stent insertion at seven RCCs. CR was performed for compensated heart failure at six RCCs. CR was performed for valvular surgery, implantation of a pacemaker or ICD, cardiomyopathy, and LVAD at three RCCs. CR was performed for aortic surgery, peripheral artery disease, and heart transplantation at two RCCs.

The annual number of AMI admissions, CR candidates, CR capacity, and CR density in the 12 RCCs are shown in Table 3. The annual number of AMI admissions, CR candidates, CR capacity, and CR density showed significant differences among the 12 RCCs. In particular, the CR capacity and CR density ranged from 50 to 500 and 0.42 to 7.36, respectively. The RCCs where CR density is more than one need to expand their CR capacity to adequately offer CR programs to each RCC’s CR candidate.

### 3.3. The CR Program’s Current Status in 12 RCCs

The CR directors at the 12 RCCs were all physiatrists (rehabilitation medicine physicians). At all RCCs, risk factor evaluation, CR evaluation, CR exercise prescription, exercise therapy under supervision, drug compliance monitoring, and CR education were conducted. Follow-up assessments were performed at 11 RCCs (92%). However, psychological evaluation (practice rate 33%), stress management (practice rate 50%), and vocational counseling (practice rate 42%) were practiced at low rates (Table 4). All RCCs were equipped with the facilities and equipment for CR. However, in some RCCs, general rehabilitation facilities and equipment were used for CR (Table 5). At most centers, the staff held concurrent positions in CR and general rehabilitation. Some personnel were even absent. Therefore, improvements were required (Table 6). At least one member of the CR staff held a license for advanced cardiac life support at all RCCs.

**Figure 2:**
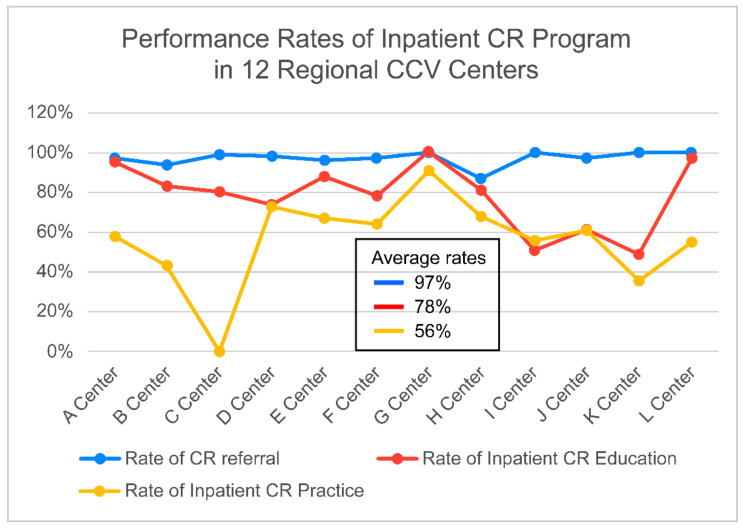
Performance Rate of Inpatient CR Program in 12 RCCs. CR: cardiac rehabilitation, RCC: Regional Cardiocerebrovascular Center.

For the CR referral method, nine centers (75%) used an electronic automatic referral system, while at three centers (25%), consultation was made at the prescription of a cardiologist or cardiac surgeon. The CR referral (97%) and patient education (78%) rates were high. However, inpatient CR exercise practice (56%) was low. There were significant gaps among the 12 RCCs (Figure 2, Table 7). One center did not operate inpatient CR exercise practices at all. There were small differences in the time to outpatient CR after PCI by RCCs. However, six RCCs (50%) began outpatient CR an average of two weeks after PCI. Most RCCs began outpatient CR four weeks after cardiac surgery. However, in five RCCs, outpatient CR for cardiac surgery patients was not practiced.

At all RCCs, most indicated that patients for CR were AMI patients and those who received PCI. However, this may be because all RCCs are annually evaluated by regular performance measures of KDCA for AMI management. According to the risk stratification for exercise-related adverse CV complications, high-risk patients participated in outpatient CR at nine RCCs. Moderate risk patients participated in CR at 11 RCCs, and even low-risk patients participated in CR at eight RCCs.

**Figure 3:**
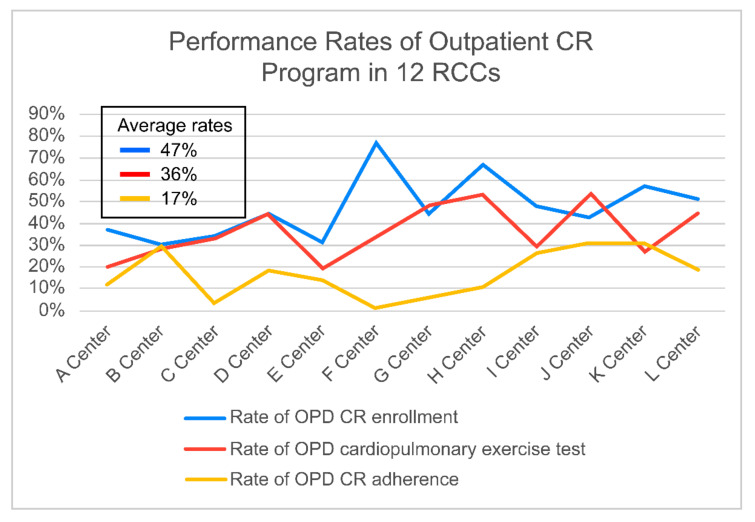
Performance Rate of Outpatient CR Program in 12 RCCs. CR: cardiac rehabilitation, RCC: Regional Cardiocerebrovascular Center.

The enrollment (47%) and adherence (17%) rates to the outpatient CR program were much lower and showed deviations among RCCs (Figure 3). Full planned session of formal out-patient CR program are 36 sessions (three times a week for 3 months). The rates of each RCC are listed in Table 8. On a Likert scale ranging from one to five, the opinions of medical staff in 12 RCCs on outpatient CR barriers were 4.1 for time/distance/transport issues. They were 3.5 for patients’ burden of CR expense, and 3.0 for a lack of recommendation to attend an outpatient CR program by a cardiologist and cardiac surgeon and a lack of CR staff (Table 9).

## 4. Discussion

CR is practiced in 111 countries globally [17], and countries including the US, Canada, Europe, Japan, and Korea have published high-quality CPG [5,8,9,10,11,12,13]. Although the level of recommendation for CR is strong, the actual CR participation rate is only about 30–40% [18]. Each nation is striving to increase the participation rate. However, this rate is only about 1.5% in Korea [19], which is much lower than those of the other aforementioned countries. In addition, there are a lack of strategies for effective management in the community after patients’ discharge and for increasing treatment compliance. The continuum of care from the hospital to the community after patient discharge is very important for determining their long-term prognosis. Therefore, a state-led system needs to be established.

A comprehensive CR program was introduced in Korea around the late 1990s. Before the establishment of RCCs, only five private medical institutions had well-organized CR programs in South Korea. They were all in densely populated areas in the capital region. Before the regional hospitals in this study were granted RCC status by the MHW, they had no CR facility, equipment, or CR specialized staff. Since 2008, the MHW of South Korea has established 14 RCCs in different provinces nationwide. This includes 12 tertiary university hospitals and two general hospitals. The intention was to reduce the medical gaps in research, knowledge, and practice for AMIs and strokes among the regions in South Korea. There may be differences in the organization of nationwide infrastructure for the CV care systems in other countries. However, it is expected that RCCs serve as key units of organization to provide nationwide quality care for CVD in South Korea. Standardized, high-quality care for AMI and stroke has been established in the RCCs. Furthermore, equipment and maintenance of CR facilities and personnel are mandatory. Many of this study’s co-authors have published articles on CR’s status and impact on AMI’s prognosis [20,21,22,23,24]. There has been an increasing acceptance of CR nationwide and an introduction of CR insurance benefits in February 2017. CR facilities have therefore increased by up to 46 centers nationwide. This includes RCCs. A growing number of medical institutions are preparing to establish CR programs. However, the CR participation rate is still very low and there are considerable differences in the CR programs’ activity levels among CR facilities [19].

According to this study’s results, considering the number of CVD patients by region, the CR demand cannot be met by CR programs in the RCCs. In Daejeon-Chungnam, Chungbuk, Jeonbuk, Ulsan, and Jeju regions, RCCs were the only hospitals practicing CR. The history of CR programs at the RCCs ranges from 2 to 13 years. Regardless of the duration, the number of AMI patients, physicians’ interest in and will to practice CR, and regional socioeconomic characteristics contributed to the CR programs’ activity levels in each RCC. The CR capacity ranged from 50 to 500. The CR density varied from 0.42 to 7.36. RCCs with a CR density of more than one, due to low CR capacity, need to expand their CR capacity to sufficiently offer CR programs to their candidates. In particular, B, C, and D centers should endeavor to urgently improve their CR capacity to reserve their standardized patient quota.

At most RCCs, the facilities, equipment, and personnel required for CR were in place. However, there were some RCCs that concurrently utilized general rehabilitation facilities and personnel for CR. This practice should be reformed. At all RCCs, risk factor evaluation, CR evaluation, CR exercise prescription, exercise therapy under supervision, drug compliance monitoring, and CR education were performed. However, psychological evaluation, stress management, and vocational counseling were practiced at low rates. These components must also be added to the comprehensive CR program for all RCCs [25,26,27].

The average RCCs’ CR referral rate was 97%. This is very high, which may be because nine RCCs (75%) used automatic referral systems. Furthermore, the medical staff reviewed the prescription for CR referral at three RCCs (25%). However, as compared to the inpatient CR referral rate, the outpatient CR participation rate was very low. The enrollment rate in (47%) and adherence to (17%) outpatient CR were much lower and showed deviations among the 12 RCCs. We are currently in the process of analyzing big data using health insurance claim data showing implementation status of CR in Korea including CR participation rate.

The factors that hinder participation may be a lack of understanding of the need for CR, lack of motivation, and various socioeconomic factors. These include time/distance/transport issues and the burden of paying CR fees. As compared to tertiary medical centers in Seoul and the metropolitan areas, the proportion of patients coming from farming/fishing villages, mountainous villages, and islands is relatively high at the RCCs. It is therefore difficult to maintain adherence to hospital-based outpatient CR. Grace et al. and a study by four institutions in Korea have reported similar barriers to CR [28,29].

Strategies are required to overcome these barriers at the patient, doctor, hospital, and policy levels [30,31]. According to this study’s results, the high average rate of CR referral (97%) is satisfactory. However, the low rate of CR enrollment (47%) and adherence (17%) to the outpatient CR programs are major challenges to be overcome regarding patient-centered strategies. First, to improve CR facilities’ accessibility, CR programs need to be established in more hospitals to form a denser CR network. According to a report on the global CR density status in 2019, the CR density in South Korea was 22. It ranked 27th out of 111 countries with CR programs [17]. Thus, RCCs should be expanded for improving the CR capacity to meet the need in their region and a regional network system should be implemented through the new “Local Cardiocerebrovascular Center (LCC)” project. This is currently being developed by the government. It not only provides timely acute medical services but also provides post discharge outcome care across the country, including rural areas [32]. For this second mission, CR programs’ installation should be made mandatory in all LCCs. The experience of CR programs in RCCs may play a central role in the establishment of CR programs in LCCs. RCCs may serve as leaders of CR networks in each region (CR hub). This may provide education and support for the LCCs and establish a CR network, where the centers may refer CR patients mutually. More flexible scheduling for outpatient CR, improvement in accessibility to CR facilities (location and direction signs) in hospitals, and more active utilization of home-based CR programs should be applied [33,34]. These include the development of standardized protocols and insurance fees, game type CR programs, and telehealth or mobile healthcare techniques. We are currently conducting a randomized controlled trials that applies hybrid CR and telerehabilitation, the strategies for improvement of CR participation, as subsequent research.

At a patient level, RCCs should provide self-efficacy counseling and education programs, psychological evaluation, and consultation to improve patients’ motivation. They could also provide specialized CR programs for elderly patients with severely reduced exercise capacity, transportation/mobility aid for the elderly, and CR participation incentives. These include reimbursement for transportation expenses or gifts for successful completion of the CR program. In addition, a CR co-pay relief program should be implemented to reduce the patients’ CR fee burden.

This study has several limitations that should be noted. First, it was based on questionnaire surveys. Since they were completed by at least one cardiologist, cardiac surgeon, or physiatrist, there is a possibility of response bias. Second, the COVID-19 pandemic adversely impacted the study’s response rate from the eligible sample. Third, since the CR in-depth questionnaires were completed by only a physiatrist, the surveys’ results may not reflect the cardiologists’ and cardiac surgeons’ perspectives. This was because the CR directors of all the RCCs were physiatrists. Future studies should include hospitals where CR directors are cardiologists or cardiac surgeons.

Despite these limitations, this study is meaningful, as it is the first study to examine and compare CR status in government-sponsored RCCs. The results showed that while RCCs are equipped with the necessary facilities, equipment, and personnel, not all the essential components are in place. More standardized clinical performance and quality measures should be used to improve the CR quality in RCCs [20,21]. The outpatient CR program is still not as active as the inpatient program. There are substantial gaps among the RCCs. Therefore, to improve CR in the RCCs, the attention and resources of the medical staff, hospital management, and standardization of the CR programs in the RCCs are required. In addition, patient-oriented CR programs should be more actively practiced to increase the rate of outpatient CR adherence. However, for the aforementioned strategies to be realized, effective governmental policy and financial support are required.

CR has been reimbursed by the Korean government since February 2017. There has yet to be a national systematic plan for improving CR implementation, and a state-controlled CR network has not yet been established as well. The activation of CR in Korea was somewhat delayed compared to other high-income countries. To overcome this situation, 12 government-driven RCCs to cover CR were established across the whole country. There are many countries where the status of CR is similar to Korea, or not yet activated. We believe it would be beneficial for such countries to refer to the situation in Korea.

Through three consecutive years of research, we intend to present strategies to improve CR participation in Korea. As the first step of this project, we investigated the current status of CR in Korea through a nationwide survey. This article, a part of the complete work, details the operating status of 12 RCCs established with government support.

## 5. Conclusions

CR in Korea began approximately 30 years after most medically advanced countries. However, the Korean KDCA realized CR’s importance and mandated the CR programs’ implementation in the RCC project. According to this study’s results, while the CR programs are in operation at an elementary level, there are improvements required. There is a large gap among the RCCs. Therefore, specific and active standardization procedures are necessary. A patient-oriented approach should be actively practiced. When appropriate improvements and financial/political support are made, the CR programs of the RCCs will be able to reach global standards.

## Figures and Tables

**Table 1:** Performance Measure Index for the CR program of RCC (Requirements of the MHW).

Performance Measure Index		Definition	Requirements
Staff	CR Director	Documentation of official certification	1
	Physician	Number of CR specialized physicians	≥1
	Physical therapist	Number of CR specialized physical therapists	≥1
	Registered Nurse	Number of CR specialized nurses	≥1
	ACLS certification	Number of CR staff with ACLS certification	≥1
Facility	CV education room	CV education room for patients and families	1
	Exercise laboratory and gym	Space dedicated for exercise test & exercise training	Area ≥ 33 m^2^
Equipment	Treadmill/Bicycle ergometer	Number of treadmills and bicycle ergometers	≥4 units
	Arm ergometer	Number of arm ergometers	≥1 unit
	CPX test equipment	Number of CPX test equipment	≥1 unit
	ECG telemetry	Number of telemetry ECG channels	≥2 units
	Emergency cart	Number of CPR emergency carts	≥1 unit
Patient data	Patient EMR data sharing	Inter-department patient EMR data sharing	Possible
Operation	Meeting for CR critical pathway	Regular meeting for practice of CR critical pathways	≥1/month
	Update and management of CP	Update of CR critical pathways and CR protocol	≥1/year
	Patient individual education	Offer individual education to CR referred patients	≥80%
	Discharge plan	Offer discharge plan for CR to patients & families	100%
	Response to CR referral	Early response to CR referral within 24 h	≥90%
		Offer submaximal exercise test to a CR referred patient	≥50%
Patient CR follow-up	3-month CR follow-up	Rate of 3 months CR F/U in CR referred patients	≥50%
	12-month CR follow-up	Rate of 12 months CR F/U in CR referred patients	≥20%
	3-month CPX test	Rate of 3 months CPX test in CR referred patients	≥30%
	6-month CPX test	Rate of 12 months CPX test in CR referred patients	≥20%
Regular meeting	CR staff meeting	Frequency of regular CR team strategy meeting	≥4/year
	National RCC meeting	National annual assembly of RCC staff	Attend

CR: cardiac rehabilitation, RCC: regional cardiocerebrovascular center, MHW: Ministry of Health and Welfare, ACLS: advanced cardiac life support, CV: cardiovascular, CPR: cardiopulmonary resuscitation, EMR: electronic medical recording, F/U: follow-up, CPX test: cardiopulmonary exercise test, including respiratory gas analysis.

**Table 2:** Number and Distribution of Hospitals Performing PCI and CR by Region in Korea.

Region ^#^	RCC	PCI Hospital *	CR Hospital **	Annul No. of IHD ^†,^^§^	Annual No. of AMI ^‡,^^§^
Seoul	0	31	13	254,753	25,715
Gyeonggi	1	33	9	184,453	23,834
Incheon	1	9	3	48,834	5926
Gangwon	1	5	2	31,748	4913
Daejeon-Chungnam	1	12	1	62,004	8204
Chungbuk	1	6	1	27,432	3254
Gwangju-Jeonnam	2 ^a^	11	2	73,777	9373
Jeonbuk	1	5	1	32,372	5091
Busan	1	17	4	84,344	8674
Gyeongnam	1	11	3	51,940	7714
Ulsan	1	5	1	17,988	2004
Daegu-Gyeongbuk	2 ^b^	15	6	93,054	15,402
Jeju	1	4	1	10,563	915
Total	14	164	47	943,006	118,872

CR: cardiac rehabilitation, RCC: Regional Cardiocerebrovascular Center. ^#^ Province or metropolitan city. * Hospitals that perform PCI (percutaneous coronary intervention). ** Hospitals with CR programs in the region, including RCC. ^†^ Ischemic heart disease. ^‡^ Acute myocardial infarction. ^§^ Data from Healthcare Big Data Hub, open data on high-interest diseases, 2019 (opendata.hira.or.kr). ^a^ 13th RCC was installed in the Jeonnam region in 2020 but was not included in this study. ^b^ 14th RCC was installed in the Gyeongbuk region in 2020 but was not included in this study.

**Table 3:** Annual AMI Admission, CR Candidate, CR Capacity, and CR Density in 12 RCCs.

RCC	AMI Admission *	CR Candidate **	CR Capacity ^†^	CR Density ^‡^
A	245	87	70	1.24
B	722	589	120	4.91
C	471	368	50	7.36
D	587	473	100	4.73
E	481	487	400	1.22
F	163	150	200	0.75
G	401	340	220	1.55
H	282	210	500	0.42
I	1170	885	300	2.95
J	145	131	50	2.62
K	362	239	240	0.99
L	271	203	300	0.80
Total	5300	4162	2350	1.77

CR: cardiac rehabilitation, RCC: Regional Cardiocerebrovascular Center. * Annual number of acute myocardial infarction admissions for each RCC. ** Annual number of CR candidates for AMI admission of each RCC. ^†^ The median number of patients, which each RCC can annually serve. ^‡^ CR candidates divided by the CR capacity of each RCC.

**Table 4:** Offered Cardiac Rehabilitation Components in 12 RCCs.

Components		No. of RCCs That Offered a CR Component That Is Relevant	
		Yes (%)	No (%)
Assessments	Cardiovascular risk factors	12 (100)	0 (0)
	Cardiopulmonary exercise test	12 (100)	0 (0)
	Other physical function test	10 (83)	2 (7)
	Assessment of comorbidity	9 (83)	3 (27)
	Psychological evaluation	4 (33)	8 (67)
	Follow-up after the end of program	11 (92)	1 (8)
	Long-term follow-up	11 (92)	1 (8)
Management	Exercise prescription	12 (100)	0 (0)
	Supervised exercise training	12 (100)	0 (0)
	Self-monitoring technique	12 (100)	0 (0)
	Stress management	6 (50)	6 (50)
Education	Risk factors control	12 (100)	0 (0)
	CV drug compliance	12 (100)	0 (0)
	Nutritional counseling	9 (83)	3 (27)
	Vocational counseling	5 (42)	7 (58)

RCC: Regional Cardiocerebrovascular Center. CV: Cardiovascular.

**Table 5:** Facility and Equipment for Cardiac Rehabilitation Program in 12 RCCs.

Facility or Equipment	No. of RCCs Holding Facility or Equipment		
	Exclusive (%)	Concurrent (%)	Absence (%)
Exercise Gym	12 (100)	0 (0)	0 (0)
Treadmill	12 (100)	0 (0)	0 (0)
Bicycle ergometer	11 (92)	1 (8)	0 (0)
Arm ergometer	5 (42)	5 (42)	2 (17)
CPX test equipment	12 (100)	0 (0)	0 (0)
Telemetry ECG monitoring system	12 (100)	0 (0)	0 (0)
Oxygen supply	12 (100)	0 (0)	0 (0)
Education room	10 (83)	2 (17)	0 (0)
Locker room	8 (67)	4 (33)	0 (0)
Resistance training equipment	8 (67)	4 (33)	0 (0)
Body composition analyzer	8 (67)	2 (17)	2 (17)

RCCs: Regional Cardiocerebrovascular Center. CPX test: cardiopulmonary exercise test. ECG: electrocardiography.

**Table 6:** Staff Composition for Cardiac Rehabilitation Program in 12 RCCs.

Staff Composition	No. of RCCs Assigned Staff Composition		
	Exclusive (%)	Concurrent (%)	Absence (%)
Director	0 (0)	12 (100)	0 (0)
Physical therapist	10 (83)	2 (17)	0 (0)
Nurse	6 (50)	6 (50)	0 (0)
Psychologist	0 (0)	4 (33)	8 (67)
Nutritionist	2 (17)	5 (42)	5 (42)

RCCs: Regional Cardiocerebrovascular Center.

**Table 7:** Rate of CR Referral, Patient Education, and Inpatient CR Program in Each RCC.

RCC	CR Referral *	Patient Education **	Inpatient CR Program ^†^
A	97%	95%	58%
B	94%	83%	43%
C	99%	80%	0%
D	98%	74%	73%
E	96%	88%	67%
F	97%	78%	64%
G	100%	100%	91%
H	87%	81%	68%
I	100%	51%	56%
J	97%	61%	61%
K	100%	49%	36%
L	100%	97%	55%
Total	97%	78%	56%

CR: cardiac rehabilitation, RCC: Regional Cardiocerebrovascular Center. * Rate of CR referral = no. of CR referrals divided by the no. of the annual CR candidates for AMI admission. ** The rate of patient education = no. of patient education divided by the annual no. for CR referral for AMI admission. † Rate of inpatient CR program = no. of the inpatient CR program divided by annual no. for CR referral for AMI admission.

**Table 8:** Rate of Outpatient CR Enrollment, Performing Baseline CPX Test, and CR Adherence.

RCC	CR Enrollment	Baseline CPX Test	CR Adherence
A	37%	20%	12%
B	30%	28%	30%
C	34%	33%	3%
D	44%	44%	18%
E	31%	19%	14%
F	77%	34%	1%
G	44%	48%	6%
H	67%	53%	11%
I	48%	29%	26%
J	43%	54%	31%
K	57%	27%	31%
L	51%	44%	19%
Total	47%	36%	17%

CR: cardiac rehabilitation, CPX test: cardiopulmonary exercise test. Rate of CR enrollment = no. of outpatient CR enrollment divided by the no. for CR referral during admission. Rate of baseline CPX test = no. of PCX test completed/no. of CR referral. Rate of CR adherence = no. of outpatient CR Completion/no. of CR referral. RCC: Regional Cardiocerebrovascular Center.

**Table 9:** Likert Scales of the Physician’s Opinion on Outpatient CR Barriers in 12 RCCs.

RCC	Lack of Recommendation *	Lack of CR Equipment	Lack of CR Space	Lack of CR Staff	Pt’s Burden of Payment	Time/ Distance/ Transport Issues **
A	3.0 ± 0.81	1.7 ± 0.47	1.7 ± 0.47	2.0 ± 0.81	3.3 ± 0.47	4.3 ± 0.47
B	3.5 ± 0.50	3.5 ± 0.50	3.5 ± 0.50	4.0 ± 0.00	3.5 ± 0.50	4.5 ± 0.50
C	4.0 ± 0.70	2.0 ± 1.00	2.0 ± 1.00	2.5 ± 1.10	4.5 ± 0.82	4.5 ± 0.86
D	2.5 ± 0.50	1.5 ± 0.50	1.0 ± 0.00	4.5 ± 0.50	4.5 ± 0.50	4.5 ± 0.50
E	1.5 ± 0.50	1.0 ± 0.00	2.0 ± 1.00	2.0 ± 1.00	2.5 ± 0.50	4.0 ± 0.00
F	3.0 ± 0.81	3.3 ± 0.47	3.4 ± 0.47	3.7 ± 0.47	3.0 ± 0.00	3.3 ± 1.24
G	3.0 ± 1.00	1.5 ± 0.50	1.5 ± 0.50	2.0 ± 1.00	3.5 ± 0.50	4.0 ± 1.00
H	4.0 ± 0.00	1.5 ± 0.50	1.5 ± 0.50	1.5 ± 0.50	3.5 ± 0.00	4.0 ± 0.00
I	3.0 ± 1.26	2.0 ± 0.89	2.4 ± 0.80	3.2 ± 0.40	3.4 ± 0.48	4.0 ± 0.89
J	2.5 ± 1.50	1.3 ± 0.43	1.8 ± 0.82	2.5 ± 1.00	3.5 ± 0.50	3.8 ± 0.43
K	4.3 ± 0.82	2.3 ± 1.29	2.5 ± 1.11	3.8 ± 0.43	3.5 ± 0.50	4.0 ± 0.70
L	3.0 ± 1.00	1.5 ± 0.50	1.5 ± 0.50	3.0 ± 2.00	3.5 ± 0.50	4.0 ± 0.00
Total	3.1 ± 1.21	2.2 ± 1.05	2.2 ± 1.12	2.9 ± 1.23	3.5 ± 0.72	4.0 ± 0.82

Values are mean ± standard deviation, Likert scale: 1—Definitely not (strongly disagree), 2—Not (disagree), 3—Neutral (neutral), 4—Yes, but minor (agree), 5—Major issue (strongly agree). CR: cardiac rehabilitation, RCC: Regional Cardiocerebrovascular Center. * Lack of recommendation to attend the outpatient CR program by a cardiologist or cardiac surgeon. ** Time, distance, or transport issues to visit a hospital for the outpatient CR program.

## Data Availability

The data that support the findings of this study are available from the Korea National Institute of Health (KNIH) but restrictions apply to the availability of these data, which were used under license for the current study, and so are not publicly available. Data are however available from the authors upon reasonable request and with permission of KNIH.

## Supplementary Materials

The following are available online at https://www.mdpi.com/article/10.3390/jcm10215079/s1, Supplementary Material S1: I. CR-GQ form, Supplementary Material S2: II. CR-IDQ form.
