# Supplementary material for: Current Status of Cardiac Rehabilitation in the Regional Cardiocerebrovascular Centers in Korea
Source: J Clin Med. 2021 Oct 29;10(21):5079. doi: 10.3390/jcm10215079 (PMC8585050; doi:10.3390/jcm10215079)
Supplement: Supplementary file 1 [file jcm-10-05079-s001.zip › Supplementary_Material_S1.pdf]

I. CR-GQ form

Reply code: ____________

You will be asked to reply to this form via an online survey.

In the case of smartphone use, once you scan the **QR code** above**,** the online survey form will be opened.
If you are using the internet, please enter **www.crsurvey.co.kr**, and select #1 – "Survey form for institutions with CR program."

1. Please check (✓) your specialty in medicine. (Please only select one option below.)
   - ☐ Cardiologist
   - ☐ Cardiac surgeon
   - ☐ Physiatrist (Boardman of rehabilitation medicine)
   - ☐ Others: ____

2. Please check (✓) all the procedures that are offered at your institution.
   - ☐ Percutaneous coronary intervention (PCI)
   - ☐ Coronary artery bypass graft (CABG) surgery
   - ☐ Percutaneous valve implantation
   - ☐ Valvuloplasty or valve replacement surgery
   - ☐ Aortic surgery (artificial vessel or stent insertion)
   - ☐ Implantable heart devices (pacemakers or defibrillators)
   - ☐ Cardiac transplant or LVAD
   - ☐ Others : ____

3. How many procedures or surgeries does your institution perform?
   1) PCI: (cases/year)
   2) CABG: (cases/year)
   3) Valvuloplasty or valve replacement: (cases/year)
   4) Admission for CHF: (cases/year)

4. In what year was your cardiac rehabilitation program initiated? _______ (year)

5. Does the hospital have an inpatient cardiology service? Please only check one box below:
   - ☐ Yes, and these patients are regularly referred to our cardiac rehabilitation program.
   - ☐ Yes, and these patients are sometimes referred to our cardiac rehabilitation program.
   - ☐ Yes, and these patients are rarely referred to our cardiac rehabilitation program.
   - ☐ No.

   5-1. Please check (✓) the CR referral methods at your institution. (Please check all that apply).
   - ☐ Automatic referral system
   - ☐ By decision of the medical staff in the cardiovascular care ward
   - ☐ Explain and suggest the CR attendance by the CR staff
   - ☐ Patient education for CR and offering related educational materials
   - ☐ The first visit to/appointment at the outpatient department of the CR before their discharge
   - ☐ Others (: ____)

   5-2. Is the CR capacity in your institution enough to supply the CR referral?
   - ☐ Yes, enough
   - ☐ No, not enough
   - ☐ Not at all

   5-3. What is the average CR referral rate at your institution? _________ %

6. In what department is the cardiac rehabilitation program administratively situated?
   - ☐ Cardiology department
   - ☐ Rehabilitation department
   - ☐ Internal medicine department
   - ☐ Cardiac surgery department
   - ☐ Others (: ____)

7. Who mainly requests CR referral in your institution?
   - ☐ Patient's request
   - ☐ Medical doctor
   - ☐ Non-physician medical personnel

☐ Others (: ____)

8. Please rate the degree to which the following are barriers to greater patient participation in your cardiac rehab program, from "this is definitely not an issue" to "this is a major issue." Please only check one option per row.

	**Definitely not**	**Not**	**Neutral**	**Yes, but minor**	**Major issue**
1) Lack of patient referral	☐	☐	☐	☐	☐
2) Lack of equipment	☐	☐	☐	☐	☐
3) Lack of space	☐	☐	☐	☐	☐
4) Lack of human resources	☐	☐	☐	☐	☐
5) Lack of financial resources/budget	☐	☐	☐	☐	☐
6) Patient's burden for CR payment	☐	☐	☐	☐	☐
7) Distance, transport problem	☐	☐	☐	☐	☐
8) Other	☐	☐	☐	☐	☐

9) Please specify other____

9. What is your opinion on effective strategies to activate the CR program in your institution?

____.
