# Supplementary material for: Current Status of Cardiac Rehabilitation in the Regional Cardiocerebrovascular Centers in Korea
Source: J Clin Med. 2021 Oct 29;10(21):5079. doi: 10.3390/jcm10215079 (PMC8585050; doi:10.3390/jcm10215079)
Supplement: Supplementary file 1 [file jcm-10-05079-s001.zip › Supplementary_Material_S2.pdf]

II. CR-IDQ form

(This survey was only for the CR staff).

1. Who has the overall responsibility for cardiac rehabilitation in your program? Please only check one box:
   - ☐ Cardiologist
   - ☐ Physiatrist (rehabilitation medicine physician)
   - ☐ Cardiac surgeon
   - ☐ Other (specify) ____

2. Which of the following components of cardiac rehabilitation are provided in your program? Please only check one box per row.

	Yes	No
Evaluation of CV risk factors	☐	☐
Cardiopulmonary exercise test	☐	☐
Assessment of strength	☐	☐
Assessment for comorbidities/issues that may impact exercise (e.g., cognition, vision, musculoskeletal/mobility issues, frailty, and/or balance/falls risk)	☐	☐
CR exercise prescription	☐	☐
Supervised CR exercise training	☐	☐
Self-assessment of exercise intensity and HR	☐	☐
Self-management of CV risk factors	☐	☐
CV drug compliance	☐	☐
Nutritional counseling and diet management	☐	☐
Psychological assessment and counseling	☐	☐
Vocational counseling/support for return-to-work	☐	☐
Stress management/Relaxation techniques	☐	☐
End of program re-assessment	☐	☐
Follow-up after outpatient program	☐	☐

3. How many patients do you have the capacity to serve each year in your institute, in terms of staff and space?
   ____________ patients per year

4. How many patients do you provide an inpatient cardiac rehabilitation program for each year in your institute?
   ____________ patients per year

5. For patients referred following a cardiac hospitalization, on average, how many weeks after their discharge does a patient begin your program? (initial outpatient CR appointments)
   1) _________ weeks after medical intervention

2) __________ weeks after cardiac surgery

6. Are there many patients waiting to attend outpatient CR programs due to exceeding the CR capacity in your institution?
   ☐ Yes
   ☐ No

7. Which types of personnel are a part of your CR team? If they are a part of your team, do they only work in CR, or do they have other departmental obligations? (Please only check one box in each row)

	Yes-only CR	Yes-partial	No
CR medical director	☐	☐	☐
Physiotherapist	☐	☐	☐
Nurse	☐	☐	☐
Psychologist	☐	☐	☐
Dietician	☐	☐	☐
Exercise specialist	☐	☐	☐
Other (specify) ______________________	☐	☐	☐

8. Does your program have each of the following items, and if yes, is its use dedicated to your program or shared with another group? (Please choose only one option in each row)

	**Dedicated**	**Shared**	**None**
**CR gym space**	☐	☐	☐
**Treadmill (running machine)**	☐	☐	☐
**Bicycle ergometer**	☐	☐	☐
**Arm ergometer**	☐	☐	☐
**CPX equipment with gas analyzer**	☐	☐	☐
**CPX equipment without gas analyzer**	☐	☐	☐
**ECG telemetry**	☐	☐	☐
**O_2 supply**	☐	☐	☐
**Education** classroom	☐	☐	☐
**Locker room**	☐	☐	☐
**Resistance training equipment**	☐	☐	☐
**Body composition analyzer**	☐	☐	☐
**Other (specify)** __________________	☐	☐	☐

9. Does your site offer a supervised Cardiac Rehabilitation program?
   ☐ Yes
   ☐ No

10. Do all your clinical staff, who supervise patients during exercise sessions, have cardiopulmonary resuscitation (CPR) training/certification?
 - ☐ Yes
 - ☐ No (skip to question 11)

10-1. If yes, are they required to renew their CPR training regularly?
 - ☐ Yes
 - ☐ No

10-2. If yes, is the CPR certification advanced or basic? (Please choose only one per row)

	ACLS	KALS	BLS
**Physicians**	☐	☐	☐
**Nurses**	☐	☐	☐
**Other**	☐	☐	☐

ACLS, advanced cardiac life support; KALS, Korean advanced life support certified by the Korean Association of Cardiopulmonary Resuscitation, BLS: Basic Life Support

Questionnaire for the outpatient supervised CR program

11. Which of the following cardiac diagnoses or indications do you accept in your supervised program? (Please check all that apply).
 - ☐ Post myocardial infarction/acute coronary syndrome
 - ☐ Stable coronary artery disease, without a recent event or procedure
 - ☐ Post percutaneous coronary intervention (PCI)
 - ☐ Post coronary artery bypass graft surgery (CABG)
 - ☐ Heart failure
 - ☐ Post valve surgery/repair or transcatheter aortic valve implantation (TAVI)
 - ☐ Heart transplant or LVAD
 - ☐ Arrhythmias (hemodynamically stable)
 - ☐ ICD/CRT or pacemaker for rhythm control
 - ☐ Congenital heart disease
 - ☐ Cardiomyopathy
 - ☐ Aortic surgery
 - ☐ Peripheral artery disease
 - ☐ Other (specify): ______________________________

12. On average, how many weeks after discharge does a patient undergo the initial cardiopulmonary exercise (CPX) test?
 - ☐ Within two weeks
 - ☐ Two-four weeks

- ☐ One month-three months
- ☐ After three months
- ☐ Not performed at all

13. On average, how many CPX tests does a patient take during the first year after discharge?
    - ☐ 1
    - ☐ 2
    - ☐ 3
    - ☐ 4-5
    - ☐ Not performed at all

14. Which of the following patient levels of cardiac risk do you accept in your supervised program? (Please check all that apply).
    - ☐ Low
    - ☐ Moderate
    - ☐ High
    - ☐ Not applicable because we do not risk stratify in our program

15. What is the standard duration of the on-site cardiac rehabilitation program provided to patients?
    __________ weeks

16. On average, how many sessions do patients come on-site for each week?
    __________ sessions per week

17. On average, how long is each exercise session (including the warm-up, aerobic exercise, strength training, and/or cool down)?
    _______________ minutes/session

18. On average, how many patients are in each exercise session?
    __________ patients / session

19. Which professionals are usually present during exercise sessions? (Please only check one box in each row)

	Yes, always	No, sometimes
CR medical director	☐	☐
Resident	☐	☐
Physiotherapist	☐	☐
Nurse	☐	☐
Exercise specialist	☐	☐

Other (specify) ______________________ ☐ ☐

---

20. Does the supervised program offer telemetry or another method of monitoring patients' clinical status while exercising? (Please check all that apply).
    - ☐ Yes, telemetry
    - ☐ Yes, other method of monitoring
    - ☐ None

    20-1. Which other method of monitoring do you use? (Please check all that apply)
    - ☐ Borg scale (perceived exertion)
    - ☐ Heart rate
    - ☐ Other (specify) ____

21. On average, how many sessions do patients actually attend outpatient supervised exercise training for?
    - ☐ Less than 10/month
    - ☐ 11-20/month
    - ☐ 21-30/month
    - ☐ More than 30/month
